# The DizzyQuest Combined with Accelerometry: Daily Physical Activities and Limitations among Patients with Bilateral Vestibulopathy Due to DFNA9

**DOI:** 10.3390/jcm13041131

**Published:** 2024-02-17

**Authors:** Erik Martin, Sofie de Hoon, Joost Stultiens, Miranda Janssen, Hans Essers, Kenneth Meijer, Wouter Bijnens, Maurice van de Berg, Nolan Herssens, Sebastien Janssens de Varebeke, Ann Hallemans, Vincent Van Rompaey, Nils Guinand, Angelica Perez-Fornos, Josine Widdershoven, Raymond van de Berg

**Affiliations:** 1 Division of Balance Disorders, Department of Otorhinolaryngology and Head and Neck Surgery, Maastricht University Medical Center+, 6229 HX Maastricht, The Netherlands; 2 School for Mental Health and Neuroscience (MHENS), Maastricht University, 6229 ER Maastricht, The Netherlands; 3 Department of Methodology and Statistics, Care and Public Health Research Institute (CAPHRI), Maastricht University, 6229 ER Maastricht, The Netherlands; 4 Department of Nutrition and Movement Sciences, NUTRIM School of Nutrition and Translational Research in Metabolism, Maastricht University, 6229 ER Maastricht, The Netherlands; 5 Research Engineering (IDEE), Maastricht University, 6229 ER Maastricht, The Netherlands; 6 Space Medicine Team (HRE-OM), European Astronaut Centre, European Space Agency, 51147 Cologne, Germany; 7 Faculty of Medicine and Health Sciences, University of Antwerp, 2000 Antwerp, Belgium; 8 Department of ENT Head & Neck Surgery, Jessa Hospital, 3500 Hasselt, Belgium; 9 Multidisciplinary Motor Centre Antwerp (M2OCEAN), University of Antwerp, 2000 Antwerp, Belgium; 10 Department of Otorhinolaryngology & Head and Neck Surgery, Antwerp University Hospital, 2650 Antwerp, Belgium; 11 Service of Otorhinolaryngology Head and Neck Surgery, Department of Clinical Neurosciences, Geneva University Hospitals, 1205 Geneva, Switzerland

**Keywords:** DizzyQuest, DFNA9, accelerometry

## Abstract

Background: DFNA9 is a genetic disease of the inner ear, causing progressive bilateral sensorineural deafness and bilateral vestibulopathy (BV). In this study, DizzyQuest, a mobile vestibular diary, and the MOX accelerometer were combined to assess the daily life functional limitations and physical activity of patients with DFNA9 suffering from BV. These parameters might be appropriate as potential candidacy criteria and outcome measures for new therapeutic interventions for BV. Methods: Fifteen DFNA9 patients with BV and twelve age-matched healthy controls were included. The DizzyQuest was applied for six consecutive days, which assessed the participants’ extent of functional limitations, tiredness, types of activities performed during the day, and type of activity during which the participant felt most limited. The MOX accelerometer was worn during the same six days of DizzyQuest use, measuring the participants intensity and type of physical activity. Mixed-effects linear and logistic regression analyses were performed to compare the DFNA9 patients and control group. Results: DFNA9 patients with BV felt significantly more limited in activities during the day compared to the age-matched controls, especially in social participation (*p* < 0.005). However, these reported limitations did not cause adjustment in the types of activities and did not reduce the intensity or type of physical activity measured with accelerometry. In addition, no relationships were found between self-reported functional limitations and physical activity. Conclusions: This study demonstrated that self-reported functional limitations are significantly higher among DFNA9 patients with BV. As a result, these limitations might be considered as part of the candidacy criteria or outcome measures for therapeutic interventions. In addition, the intensity or type of physical activity performed during the day need to be addressed more specifically in future research.

## 1. Introduction

The vestibular system facilitates postural control, gaze stabilization, and spatial orientation [1,2]. In patients with bilateral vestibulopathy (BV), vestibular function is bilaterally impaired. Therefore, the most commonly reported symptoms of BV are imbalance and oscillopsia (a sensation of blurred vision during head or body movements due to the loss of gaze stabilization) [3,4]. Other known symptoms range from vertigo and dizziness to emotional and cognitive symptoms, such as anxiety, depression, and memory or attention deficits [5,6]. BV is a chronic condition resulting from many different etiologies, varying from ototoxicity, auto-immunity and neurodegeneration, to genetic mutations [7,8]. One of these genetic causes is DFNA9, an autosomal-dominant inherited mutation in the COCH gene domains homologous to coagulation factor C domains [9]. This genetic disease of the inner ear results in adult-onset progressive loss of cochleovestibular function, causing bilateral sensorineural deafness and BV [10,11]. While deafness can be treated with hearing aids or a cochlear implant [12], unfortunately, no effective treatment is available for vestibular loss as present in DFNA9 or BV in general. Therefore, the treatment of vestibular loss is nowadays mainly restricted to balance training and counselling. However, the use of vestibular implants is currently being investigated [13,14].

As previously mentioned, DFNA9 is associated with progressive vestibular loss, often resulting in BV, which is known to reduce physical activity and to increase functional limitations [15]. These activities and limitations include, among others, situations that involve standing, walking, cycling or when making fast head movements [16]. Furthermore, a decline in cognitive reserve was found in BV patients, compromising the performance of dual tasks or complex tasks [17]. These physical, functional, and cognitive challenges can lead to a progressive reduction in professional and social participation, which might negatively affect quality of life [18,19]. As a consequence, the improvement of mobility was the most prominent wish expressed by patients who were interviewed about treatment expectations [20].

New therapeutic interventions for BV are currently being investigated. Therefore, it is necessary to establish appropriate candidacy criteria and outcome measures for therapeutic interventions. Physical activity and functional limitations could be considered as potential candidacy criteria or outcome measures, since they can significantly impair quality of life in BV patients [18,20,21]. However, there is no gold standard for testing physical activity and functional limitations [22,23]. Many options can be considered, including accelerometers, pedometers, heart rate monitors, indirect calorimetry, and direct observation, as well as physical activity diaries and recall questionnaires [24]. Unfortunately, no strategy provided solid results and external validity was limited [25,26]. Recently, the use of wearable devices to measure physical activity has gained increased attention. In contrast to other approaches, such as video-analysis or laboratory-based systems, wearable accelerometers can be used in the flow of daily life, increasing the ecological validity of the obtained results. One of these wearables is the MOX accelerometer (MOX; Maastricht Instruments, Maastricht, The Netherlands), which has been demonstrated to accurately measure physical activity [27,28]. Moreover, a new app-based vestibular diary was recently introduced by the DIZZYNET Network that may be able to capture functional limitations: The DizzyQuest [29]. This diary investigates self-reported neuro-otological symptoms and their psychosocial context. It uses the experience sampling method in which questionnaires are distributed multiple times a day at semi-random moments in the flow of daily life. This strategy reduces the risk of recall bias and increases the ecological validity of the obtained results [30,31,32,33]. Therefore, combining the DizzyQuest with the MOX accelerometer might provide useful insights into the functional limitations and physical activity of patients as present in their daily lives. In the future, these tools may be considered as part of the candidacy criteria or outcome measures for therapeutic interventions in BV.

The main objective of this study is to combine the DizzyQuest with the MOX accelerometer and, therefore, use subjective and objective measurements to investigate the daily life functional limitations and physical activity of DFNA9 patients suffering from BV. It was hypothesized that DFNA9 patients with BV experience a higher extent of functional limitations and tiredness during the day and, therefore, lower their physical activity and alter the type of daily activities compared to the control group of the same age.

## 2. Materials and Methods

### 2.1. Study Population and Procedures

Patients with BV due to DFNA9 were recruited from the Department of Otorhinolaryngology and Head and Neck Surgery of Maastricht UMC+ and from “De Negende Van” foundation. The latter is a foundation for patients suffering from DFNA9. Recruitment started in September 2019 and ended in April 2020. Inclusion criteria comprised: (1) diagnosed with DFNA9; (2) diagnosed with BV according to the diagnostic criteria of the Bárány Society (see “Vestibular testing”) [34]; (3) aged 18 years or older; (4) in possession of a smartphone or tablet to download and use the DizzyQuest application; and (5) having a good understanding of the Dutch language. Patients with a history of neurological diseases, such as cerebellar ataxia, were excluded.

An age-matched control group was recruited using community-based advertisements. Inclusion criteria comprised: (1) age-matched with the patient group; (2) in possession of a smartphone or tablet to download and use the DizzyQuest application; and (3) having a good understanding of the Dutch language. Participants were screened for vestibular disorders and other physical impairments by mail or phone. In the case of a vestibular disorder or physical impairment, they were excluded from the study.

### 2.2. Vestibular and Audiometric Testing

Previous clinically obtained vestibular test results were included in this study. Therefore, vestibular testing was performed at four different centers in the Netherlands and Belgium: Maastricht University Medical Center, Gelre Hospitals, Radboud University Medical Center, and Antwerp University Hospital. Depending on the center, the patients underwent a video head impulse test and/or a caloric test to confirm the BV diagnosis according to the Bárány criteria. These criteria include: symptoms of imbalance when standing or walking, and/or movement-induced oscillopsia during walking or head movements, and a bilaterally reduced bithermal caloric response (mean peak slow phase velocity < 6°/s), and/or a bilaterally reduced vestibular–ocular reflex gain of <0.6 measured with the video head impulse test or <0.1 measured with the torsion swing test [34].

Video head impulse testing was performed in three centers: Maastricht University Medical Center, Gelre Hospitals, and Antwerp University Hospital. The video head impulse test was performed with the patients sitting on an immobile chair to prevent unwanted body movements during the test. Patients were instructed to visually fixate on a target at 1.5 m in front of the chair, while the technician applied fast (>150°/s peak velocity) and unpredictable head movements (impulses) in the horizontal plane. Head and eye movements were captured and analyzed by video head impulse test devices from Otometrics (Natus, Taastrup, Denmark). Video head impulse test gains of the lateral semicircular canals were used as an outcome parameter.

The caloric test was performed in three centers: Maastricht University Medical Center, Radboud University Medical Center, and Antwerp University Hospital. In these three centers, the caloric test was performed in a completely dark room, with the subjects in the supine position and with their head elevated 30° from the horizonal plane. Bithermal caloric irrigations were used with temperatures of 44 °C (warm) and 30 °C (cold). The type of irrigations (water or air) and eye movement recording differed between the centers. The Maastricht University Medical Center used water irrigations and eye movements that were recorded with electronystagmography (Kingslab 1.8.1, Maastricht University, Maastricht, the Netherlands). Antwerp University Hospital used air irrigation with temperatures ranging between 47 °C (warm) and 26 °C (cold) for 30 s [35]. Eye movements were recorded using videonystagmography (Otometrics ICS Chart 200 VNG, Natus, Taastrup, Denmark). The Radboud University Medical Center used different paradigms for vestibular testing. Caloric testing was performed using water irrigations for 30 s or air irrigations for 60 s and eye movements were recorded with electronystagmography (Difra Instruments Welkenraedt, Eupen, Belgium).

Finally, all DFNA9 patients underwent audiometric testing. The pure tone average (PTA) of octave frequencies at 0.5–4 kHz was used as an outcome parameter and measured in decibel (dB) hearing loss (HL). 

### 2.3. DizzyQuest

The DizzyQuest is an app-based diary designed for patients with vestibular disorders, capturing neuro-otological symptoms and their psychosocial context [31]. It runs from the UM-ESM platform, which is an experimental version of the PsyMate™ app (www.psymate.eu, accessed on 1 October 2019). It provides the opportunity to implement multiple questionnaires and sampling schemes. The DizzyQuest can be downloaded for devices running on iOS and Android (www.dizzyquest.com, accessed on 3 August 2020). Although this vestibular diary consists of four different questionnaires, only two questionnaires were used in this study: the within-day and evening questionnaire. Both questionnaires consist of a specific set of questions regarding neuro-otological symptoms and their psychosocial context (see Supplementary Materials S1 for all questions and their response options). The within-day questionnaire appears at multiple semi-random moments each day (one questionnaire within each 1.5 h interval) and investigates the current situation (measuring “in the moment”). It, therefore, avoids recall bias. The evening questionnaire reflects on the symptoms of the past day and serves as an end-of-day diary. The time to complete one questionnaire is two minutes on average [31].

### 2.4. The MOX Accelerometer

**Figure 1:**
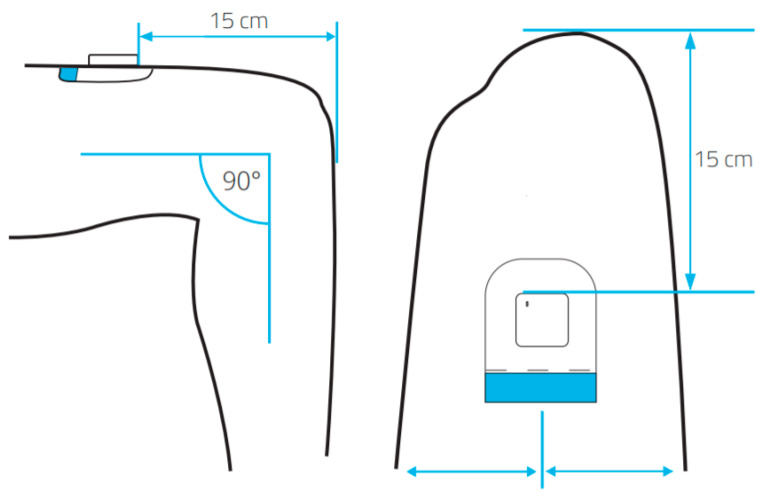
Location of the MOX accelerometer on the upper leg.

The MOX accelerometer (MOX; Maastricht Instruments BV, Maastricht, The Netherlands) measures the physical activity over three orthogonal sensor axes, including anterior–posterior, medio–lateral, and vertical, with a range of approximately 8 times gravity. These raw data are stored directly on the internal memory, with a 25 Hz sampling rate. As shown in Figure 1, a specifically designed plaster was used to place the MOX at the upper leg. The removal of the accelerometer during sleep or when taking a shower was unnecessary due to the body placement and waterproof design [28]. The MOX can be used up to seven consecutive days.

An algorithm classifies the raw acceleration of the MOX into five physical activity classifications: sedentary (sitting/lying down), standing, light physical activity (LPA), moderate physical activity (MPA), and vigorous physical activity (VPA). This algorithm uses a decision tree classifier, which divides the raw data into sedentary, standing, or dynamic behavior [26]. These raw data are pre-processed and segmented into one-second long windows using a fixed non-overlapping sliding window [36]. Using the signal magnitude area (SMA), the amount of activity for each window determined is expressed as counts per second [26,37]. Based on this value, each window is classified as static or dynamic. Dynamic windows with an amount of activity up to eight counts per second were classified as LPA, amounts of activity from eight to sixteen counts per second as MPA, and amounts of activity from sixteen counts per second as VPA. In addition to these classifications, the daily intensity of daily physical activity is calculated as mean of the counts per minute for 24 h.

### 2.5. Study Design

All participants received login codes and an instructional video on how to install and use the DizzyQuest application. After the installation, the application was used for seven consecutive days, always starting on a Monday. The participants were alerted to the questionnaire by a notification on their phone. The within-day questionnaires appeared at ten semi-random moments each day, between 7:30 a.m. and 10:30 p.m. After the questionnaire appeared, the participant had fifteen minutes to answer the questions. The evening questionnaires appeared at 7 p.m. and remained available until 4 a.m. of the following morning. The MOX accelerometer was worn during the same seven consecutive days of DizzyQuest use. The accelerometers were set up to start measuring physical activity at 0:00 p.m. on day one and measurements ended seven days later at the same time.

### 2.6. Data Selection

Participants were included for analysis if, for a minimum of two days, at least one within-day questionnaire or evening questionnaire was answered and MOX data were obtained during the same days. DizzyQuest data from day 1 were not included in the analysis, since the first day was used for the participants to become used to the DizzyQuest application and questionnaires. Therefore, a maximum of six evening questionnaires (6 days × 1 questionnaire) and 60 within-day questionnaires (6 days × 10 questionnaires) were available for analysis per participant. From the within-day questionnaire, two questions were selected for analysis: (1) I feel tired? and (2) What am I doing? (just before receiving the notification). The questions included from the evening questionnaires were: (1) I generally felt tired today, (2) to what extent were you limited in your activities today, and (3) in which type of activities were you limited the most today? These questions were selected based on their relevance in patients with BV [20] and their close correlation with (possible limitations in) physical activity. The questions were selected in consensus by two of the authors (SdH and RvdB). Regarding data from the MOX accelerometer, days containing data for over four hours continuous zero input were considered as missing input and were excluded from the analysis.

### 2.7. Data Analysis

**Figure 2:**
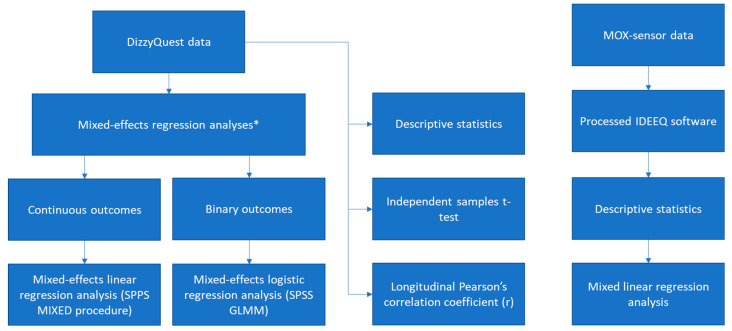
Flowchart of the data analysis. * In the case of a quadratic relationship, quadratic independent terms were added to the model. Different nested mixed models with and without serial correlation were compared in order to find the most parsimonious model.

DizzyQuest data were exported from the DizzyQuest database and accelerometry data were exported from IDEEQ (Maastricht Instruments, Maastricht, The Netherlands) to IBM SPSS Statistics 25 (IBM Corporation, New York, NY, USA). SPSS was used to analyze the data. Figure 2 shows an overview of the data analysis.

To control for the hierarchical, two-level structure of the data, with measurements (level-1) clustered within subjects (level-2) mixed-effects regression analyses were applied. To compare DFNA9 patients and the controls with respect to continuous outcomes, mixed-effects linear regression analysis was conducted using the MIXED procedure in SPSS. In the case of binary outcomes, mixed-effects logistic regression analysis was performed using the SPSS option Generalized Linear Mixed Models (GLMM). These mixed-effects models account for the fact that multiple responses from the same person are more similar than responses from other people. Different nested mixed models with and without serial correlation were compared in order to find the most parsimonious model [38]. The control group was considered as the reference group. In the case of a quadratic relationship, quadratic independent terms were added to the model. To determine the relationship between the self-reported functional limitations or tiredness and physical activity, the longitudinal Pearson’s correlation coefficient (r) was calculated as the median of the six Pearson’s r values per day. An independent samples t-test was performed to calculate the estimated difference in the response rates for the within-day and evening questionnaires between the DFNA9 patients and the control group. The significance level was set at 0.05.

The extent of limitations obtained from the evening questionnaires was calculated as mean score per participant. These mean scores were used as input to calculate a total mean score of limitations per day for both groups: DFNA9 patients and the controls. The extent of tiredness was extracted from the within-day questionnaires. Since multiple answers a day per participant could be present (the within-day questionnaire was sent 10 times a day), the mean score was first calculated per participant per day. These mean scores were then used to calculate the total mean score per group per day. The results regarding the mean extent of limitations and the mean extent of tiredness per group were both visualized over the six consecutive days, including error bars indicating the measured standard deviation.

Two items regarding activities were visualized using pie charts: (1) the type of activities being performed by the participants and (2) the activities in which the participants felt most limited. Pie charts were constructed using the mean reported frequency of each activity for both items. For each subgroup (DFNA9 patients with BV and the controls), the mean reported frequency of each activity was determined by calculating the subgroup mean of the mean of all (mean) daily scores per participant. Differences in the activities between the DFNA9 patients and control group were tested merely for the activities with the largest difference between the subgroups.

Accelerometry data were first processed in IDEEQ. The mean intensity of physical activity of the participants was first calculated per participant per day. These mean scores were used to calculate the mean intensity of the patients and control group and visualized over the six days, including error bars illustrating the standard deviation. For the distribution of the classification of physical activity during the day, first the frequency per classification of physical activity was calculated per participant per day. These frequencies were used to calculate the mean frequency per classification for the total six days per participant. Then, the obtained frequencies were used to calculate the mean frequency per classification of physical activity for both subgroups. These mean frequencies of the physical activity classification were visualized using bar charts, including error bars to illustrate the standard deviation.

To investigate whether the self-reported functional limitations and tiredness were related to mean physical activity intensity, the mean extent of functional limitations or tiredness and physical activity intensity per day per participant was used. Both the extent of functional limitations and tiredness were selected from the evening questionnaires.

Different parameters were calculated to explore potential differences in physical activity patterns between both groups: (1) number of transitions between periods of rest (sedentary and standing) and activity (LPA, MPA, and VPA); (2) duration of periods of rest and activity; and (3) distribution of mean physical activity intensity throughout the day [39,40]. Periods of rest and activity were counted in minutes per day. Mean intensity per hour was calculated as the average intensity of 60 min, resulting in 24 periods per day. The number of transitions between periods of rest and activity and duration of periods of rest and activity were visualized using the mean of each group per day. The distribution of mean physical activity intensities were visualized by calculating, for each patient per period, the mean intensity of all days. This was used as input to calculate for each group the mean of the means per period, plotted at 24 separate periods, representing one “average” day per group.

### 2.8. Ethical Considerations

Approval for this study was obtained from the research ethics board of Maastricht University Medical Center (protocol: METC 2018-0809). Informed consent was given prior to participation through the DizzyQuest application.

## 3. Results

### 3.1. Patient Characteristics and Response Rates

Thirty-three participants were included in this study, of which six participants experienced problems using or downloading the DizzyQuest application or wearing the MOX accelerometer. Therefore, twenty-seven participants were eligible to be included in the data analysis. This included fifteen DFNA9 patients with bilateral vestibulopathy (BV) and twelve age-matched controls. Table 1 shows the DFNA9 patient characteristics and the results of vestibular testing and audiometry. The DFNA9 group consisted of four males and eleven females with a mean age of 62 years (range of 50–75 years). The mean pure tone average of the DFNA9 patients was 45 dB HL (range of 5 dB–74 dB HL). The control group consisted of four males and eight females, with a mean age of 58 years (range of 46–75 years).

The mean average response rate of the DFNA9 patients was six times a day (range of 3–9 times a day) for the within-day questionnaire and six times during the week (range of 2–6 times during the week) for the evening questionnaire. The control group responded with an average of six times a day for the within-day-questionnaire (range of 3–8 times a day) and six times during the week (range of 5–6 times during the week) for the evening questionnaire. The response rates of the within-day and evening questionnaires did not differ significantly between the DFNA9 patients and the control group (−2.0 × 10^−4^ 95% CI [−0.17974, −0.17935], t(25) = −0.002, *p* = 0.998).

### 3.2. Subjective Measurements of Functional Limitations and Tiredness

**Figure 3:**
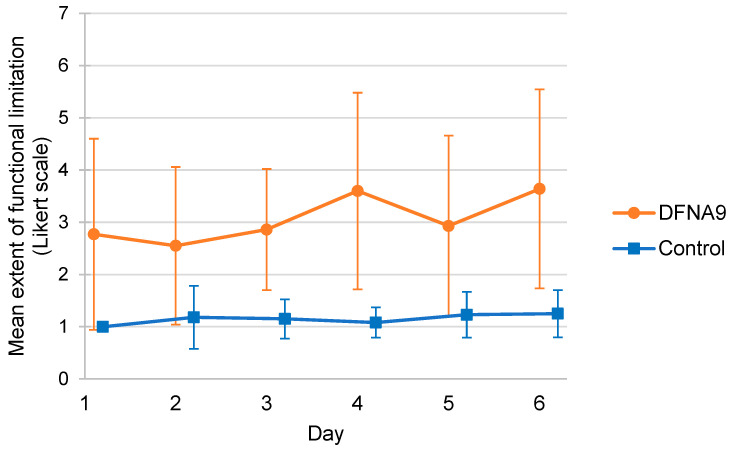
Mean self-reported extent of functional limitations of DFNA9 patients with BV and the age-matched control group, measured during six consecutive days. The higher the number on the Likert scale, the higher the experienced functional limitation (1 = not at all; 7 = very much). Dots and squares represent the mean of groups; error bars indicate standard deviation.

Figure 3 illustrates the mean self-reported extent of functional limitations of DFNA9 patients with BV and the age-matched control group, measured during six consecutive days. It can be observed that the group of DFNA9 patients with BV, in general, experienced more functional limitations than the control group. The latter group reported almost no functional limitations. Variability in the reported functional limitations was also higher in the group of DFNA9 patients with BV. Mixed linear regression analysis showed a significantly higher reported Likert score for functional limitations in DFNA9 patients with BV compared to the control group (B = 1.985, t(24.947) = 4.310, *p* < 0.005). On average, DFNA9 patients with BV scored 1.985 points higher on the Likert scale regarding functional limitations (95% CI [1.102, 2.912]) compared to the control group.

**Figure 4:**
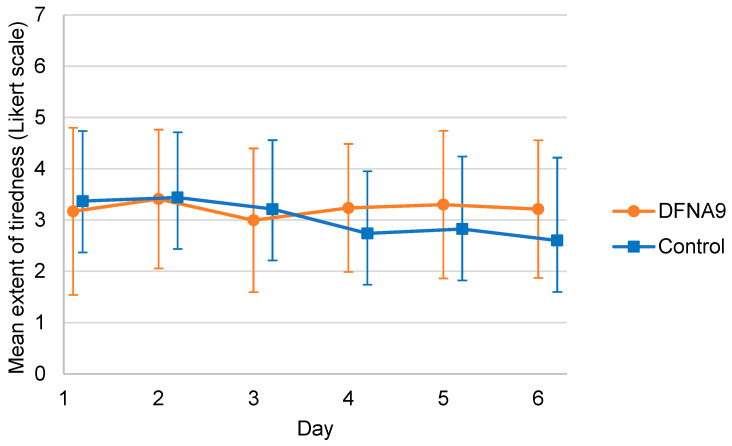
Mean of individual mean self-reported extent of tiredness of DFNA9 patients with BV and the age-matched control group, measured during six consecutive days. The higher the number on the Likert scale, the higher the experienced functional limitation (1 = not at all; 7 = very much). Dots and squares represent the mean of groups; error bars indicate standard deviation.

Figure 4 presents the mean of the individual mean self-reported extent of tiredness of DFNA9 patients with BV and the age-matched control group, measured during six consecutive days. Variability in the reported tiredness among the participants was present in both groups. The group of DFNA9 patients with BV showed higher mean scores regarding the extent of tiredness in six days compared to the control group. However, the mixed linear regression analysis did not show a significant difference in the self-reported extent of tiredness between both groups (B = 0.614, t(25.029) = 1.528, *p* = 0.139).

### 3.3. Subjective Measurements of Performed Activities and Activities during Which Participants Felt Limited

**Figure 5:**
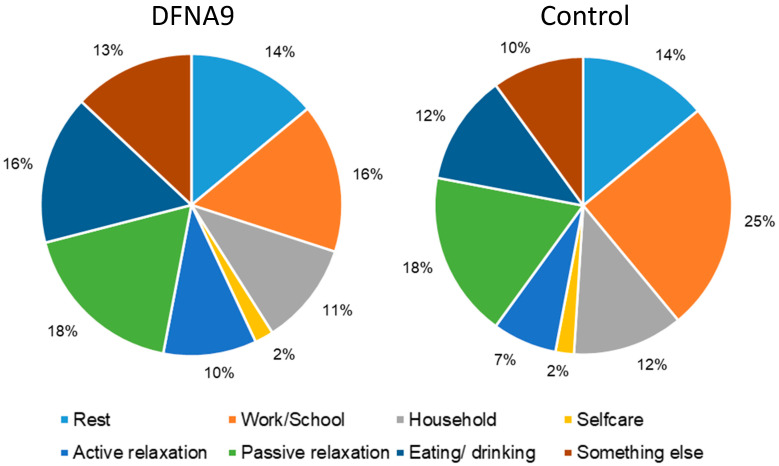
Relative frequency (%) of the type of activities performed during six days for patients with DFNA9 with BV and the age-matched control group.

Figure 5 demonstrates the relative frequency (%) of the type of activities performed during six days for patients with DFNA9 with BV and the age-matched control group. The data were obtained from the within-day questionnaire. The figure illustrates that proportions of performed activities were very similar between both groups. DFNA9 patients with BV reported being more occupied with “eating/drinking” and “something else”, while the control group reported to be more occupied with “work/school”. There was no significant difference in the probability of being occupied with work/school among the subjects in the DFNA9 patient group compared with the control group (OR = 0.386, *p* = 0.137, 95% CI [0.106, 1.405]), as shown by the mixed logistic regression analysis.

**Figure 6:**
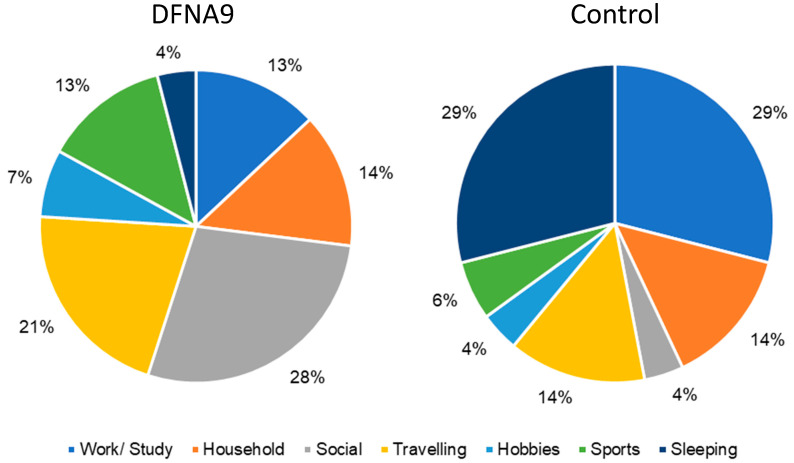
Relative frequency (%) of the type of activities during which participants felt most limited during the day (obtained during six consecutive days) for patients with DFNA9 with BV and the age-matched control group.

Figure 6 presents the relative frequency (%) of the type of activities during which participants felt the most limited during the day (obtained during six consecutive days) for patients with DFNA9 with BV and the age-matched control group. It needs to be noted that the severity of the limitation was not taken into account. It can be observed that DFNA9 patients reported being most limited in social participation and travelling. The mixed logistic regression analysis revealed that the probability of being limited in social participation was significantly higher in this group compared to the control group (OR = 10.174, *p* = 0.006, 95% CI [2.127, 48.674]). This was not significant for travelling (OR = 1.868, *p* = 0.361, 95% CI 0.466, 7.481]). The likelihood of being limited in sleeping was significantly less in DFNA9 patients compared to the controls (OR = 0.086, *p* = 0.014, 95% CI [0.013, 0.579]).

### 3.4. Objective Measurements of Physical Activity

**Figure 7:**
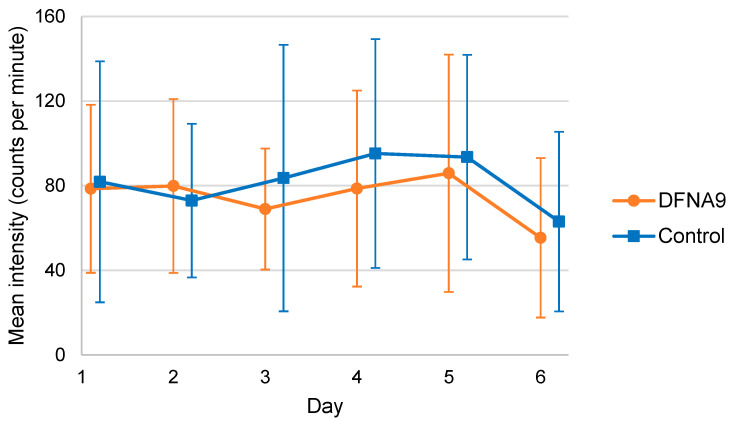
Mean of individual mean physical activity intensities per day for DFNA9 patients with BV and the age-matched control group, obtained during six consecutive days. The higher the intensity (more counts per minute), the more vigorous the activity. Dots and squares represent the mean of groups; error bars indicate standard deviation.

Figure 7 demonstrates the mean of individual mean physical activity intensities per day for DFNA9 patients with BV and the age-matched control group, obtained during six consecutive days. Variability in physical activity intensity among the participants was present in both groups. In four out of six days, the mean physical activity intensity was higher in the control group. However, the mixed-effect linear regression analysis did not show any significant difference between the mean physical activity intensities of both groups (B = −13.478, t(25.402) = −0.993, *p* = 0.330).

**Figure 8:**
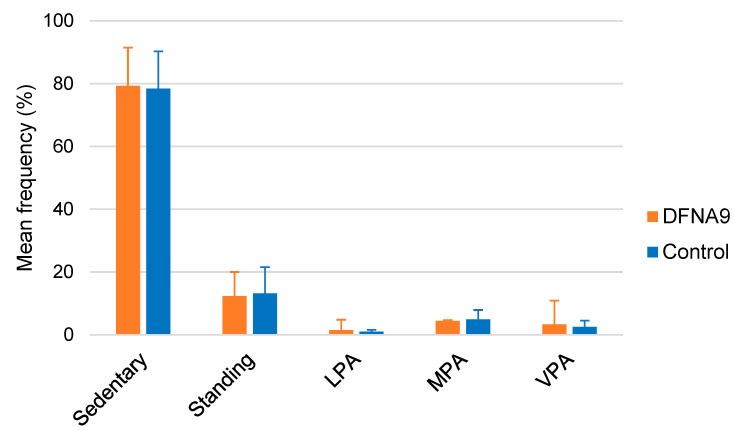
Mean distribution of the type of physical activities performed by DFNA9 patients with BV and the age-matched control group, during six consecutive days. Error bars indicate standard deviation. LPA: low physical activity; MPA: moderate physical activity; VPA: vigorous physical activity.

Figure 8 illustrates the mean distribution of the classification of physical activities performed by DFNA9 patients with BV and the age-matched control group, during six consecutive days. It can be observed that activities classified as “sedentary” were most present in both groups. Both groups did also not differ significantly regarding the distribution of classification of activities, as determined by the mixed linear regression analysis (*p* ≥ 0.425).

### 3.5. Self-Reported Functional Limitations and Tiredness Related to Mean Physical Activity Intensities

A negligible to poor correlation was found between daily self-reported functional limitations or tiredness and daily mean physical activity intensities (r = −0.064 *p* = 0.917 and r = −0.178, *p* = 0.075, respectively).

### 3.6. Comparison of Activity Patterns

**Figure 9:**
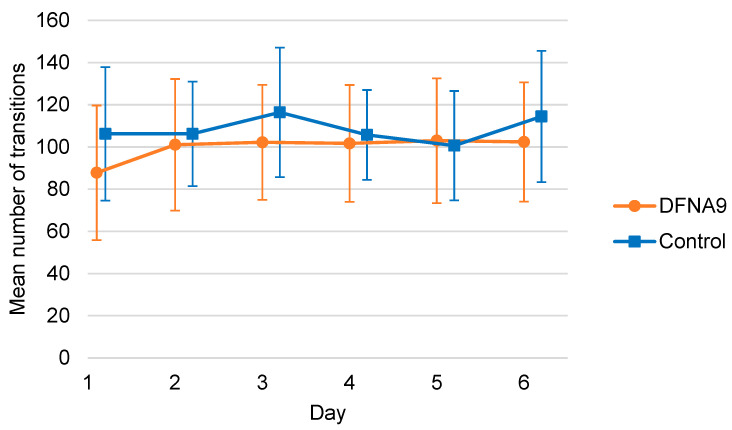
Mean number of transitions between periods of rest and activity for DFNA9 patients with BV and the age-matched control group, obtained during six consecutive days. Dots and squares represent the mean of groups; error bars indicate standard deviation.

Figure 9 shows the mean number of transitions per day between periods of rest and activity for DFNA9 patients with BV and the age-matched control group, during six consecutive days. The control group showed, on average, more transitions than the patients for most of the days. However, this difference was not significant as determined by the mixed linear regression (B = −10.350, t(25.043) = −1.165, *p* = 0.255).

**Figure 10:**
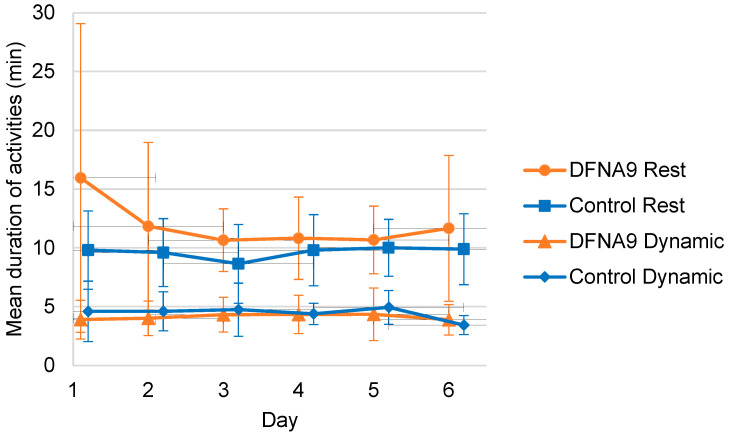
Mean duration of periods of activity and rest for DFNA9 patients with BV and the age-matched control group, obtained during six consecutive days. Dots and squares represent the mean of groups; error bars indicate standard deviation.

Figure 10 presents, for both groups, the mean duration of periods of activity and rest per day, obtained during six consecutive days. It can be observed that DFNA9 patients with BV demonstrated on average shorter periods of activity and longer periods of rest. However, these differences were not significant (B = −0.092, t(25.291) = −0.209, *p* = 0.836 and B = 1.939, t(22.609) = 1.567, *p* = 0.131, respectively).

**Figure 11:**
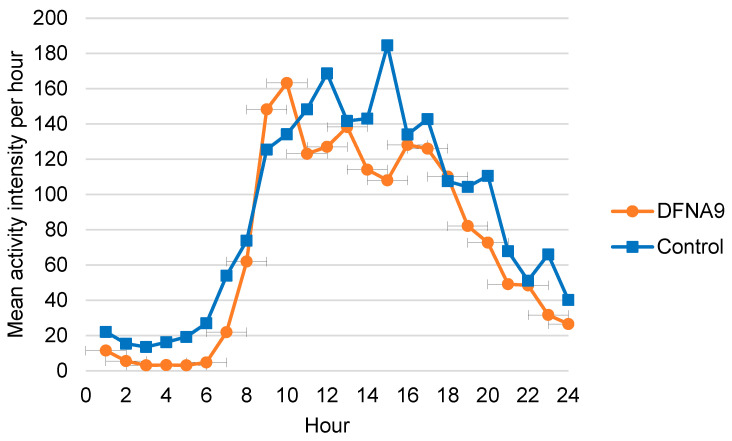
Distribution of the mean of individual mean physical activity intensities per 24 h for DFNA9 patients with BV and the age-matched control group, obtained during six consecutive days. The higher the intensity (more counts per minute), the more vigorous the activity. Dots and squares represent the mean of groups.

Figure 11 illustrates for both groups the distribution of the mean of individual mean physical activity intensities per hour for 24 h, obtained during six consecutive days. The means of individual mean physical activity intensities were generally lower during the afternoon in DFNA9 patients with BV compared to the controls. No significant difference was found between the two groups regarding the distribution of mean physical activity intensity per day (B = −16.226, t(24.887) = −1.286, *p* = 0.210).

## 4. Discussion

The aim of this study was to investigate daily functional limitations and physical activity in DFNA9 patients with bilateral vestibulopathy (BV) compared to age-matched controls using the combination of the DizzyQuest application and the MOX accelerometer. It was found that DFNA9 patients with bilateral vestibulopathy felt significantly more limited during daily activities than the age-matched controls, especially regarding social participation. However, contrary to the hypothesis of this study, this was not reflected in the type of activities or the amount or type of physical activities performed during daily life. Furthermore, no relationships between self-reported functional limitations and physical activity intensities were found.

This study demonstrated that DFNA9 patients with BV experience significantly more functional limitations during daily life than the control group. This is congruent with previous qualitative research involving face-to-face interviews, in which it was shown that the physical, cognitive, and emotional symptoms resulting from BV can lead to functional limitations when performing activities [16,20]. It also illustrated that 67% of BV patients report tiredness as a symptom of BV. In this study, DFNA9 patients with BV did not seem to suffer significantly more from tiredness than the control group [15,16]. Different reasons could be hypothesized that might explain this possible paradox. First, the previous qualitative literature did not take into account a control group. It is, therefore, unknown which percentage of a control group would have indicated tiredness as a complaint in that specific setting. This, however, does not rule out tiredness as a significant symptom of BV. After all, the investigated disorder (DFNA9) might also account for this finding. DFNA9 is a chronic slowly progressive genetic disorder. Patients suffering from chronic and slowly progressive disorders can adapt well to their symptoms over time, due to shifts in their frame of reference [16,41]. Therefore, tiredness might still be present as a symptom, but patients might consider it less of a burden due to adaptation. Finally, questionnaires (such as DizzyQuest) are less sensitive in capturing adaptive processes compared to interviews in which more follow-up questions are asked [42]. In conclusion, the self-reported functional limitations could be considered as part of the candidacy criteria and/or outcome measures for therapeutic interventions for BV. However, self-reported tiredness might not be as suited as a candidacy criterion or outcome parameter, although this could depend on the patient population or the questioning technique.

DFNA9 patients with BV mainly reported being limited in activities regarding social participation and travelling. This corresponds well with the results obtained in previous studies, in which BV patients reported difficulties in social participation due to problems with travelling [16,19,21]. The age-matched control group mainly reported limitations with respect to sleeping. However, it should be noted that the severity of the limitation was not taken into account. After all, most participants of the control group did not report significant limitations. Since the question “In which type of activities were you limited the most today?” involved a forced choice between activities, participants were not able to skip this question in the case that no limitations were experienced that day. The modification of this question (e.g., the opportunity to “skip” the question) could be considered. Therefore, a comparison between DFNA9 patients with BV and the control group regarding this question was of little value. Nevertheless, it can be concluded that this study was able to reproduce the results of previous studies regarding limitations in specific types of activities, using data captured by the DizzyQuest application. Furthermore, no significant difference was found between DFNA9 patients with BV and the age-matched control group regarding the type of activities performed during daily life. This is congruent with the previous literature and could possibly be explained by the fact that daily activities are complex, but practiced multiple times a day over the course of a lifetime, and therefore well-learned and not necessarily avoided [43]. It can, therefore, be hypothesized that the activities in which patients are limited might be considered as part of the candidacy criteria and/or outcome measures for intervention, but the type of performed activities could be less relevant.

Mean physical activity intensities, the classification of physical activities (e.g., sedentary behavior and vigorous physical activity) and activity patterns [39,44,45,46] did not significantly differ between DFNA9 patients with BV and the age-matched control group. This might be explained by the fact that this study focused on the frequency of activity intensity and the frequency of transitions between physical activity, rather than on the duration of single epochs of activity [40]. However, the frequency of different physical activity types, the number of transitions between them, and the mean duration of activity between both groups did not differ, indicating no shorter periods of activity in one group. Conflicting evidence exists in the literature regarding these findings. Although unilateral vestibulopathy patients demonstrated significantly more sedentary behavior and performed significantly less light and total physical activity compared to the control group [47], this was not confirmed in patients with BV [48]. Furthermore, the results of this study are partially congruent with recent findings, which showed that patients with early stage neurological gait disorders (including peripheral vestibular disorders) were found to maintain their everyday routine, and only a significant reduction in the total amount of ambulatory activity was demonstrated [40]. This reduction in ambulatory activity was not found in this study. However, due to the smaller sample size of this study, a potential difference between DFNA9 patients with BV and the control group regarding this parameter cannot fully be excluded. Taken all these results into account, it can be concluded that the amount and classification of physical activities performed during daily life could be addressed more specifically in future research.

Overall, no relationship existed between the extent of functional limitations or tiredness and the intensity of physical activity. This might be related to interpersonal differences in coping mechanism and symptoms experience [49,50]. After all, self-reported symptoms are influenced by psychosocial and environmental factors, which might modify perception.

### Limitations

In this study, four main limitations can be identified. First, participants from the age-matched control group did not undergo any vestibular testing or audiometry. As a result, it cannot be ruled out that some participants from the control group suffered from a vestibular disorder. To minimize this, a questionnaire was distributed to screen for vestibular disorders and exclude participants with a possible vestibular disorder. Nevertheless, in the case that participants of the control group suffered from an undetected vestibular disorder, this study could potentially have underestimated the effects between DFNA9 patients with BV and the control group. Secondly, this study only included patients with DFNA9. Therefore, results might not reflect the whole BV population in general. After all, DFNA9 is a slowly progressive disorder that might facilitate adaptation to symptoms in contrast to other clinical pictures found in BV (e.g., rapidly progressive and BV with recurrent vertigo) [3,19,43,51]. For this reason, our study results cannot be extrapolated to the BV population in general. Furthermore, DFNA9 patients do not seem to experience as much psychological distress as found in the general BV population [16,20]. DFNA9 is also characterized by sensorineural hearing loss. It is likely that hearing loss partially contributed to the self-reported functional limitations in this study [52,53,54]. Thirdly, a selection bias might have (partially) influenced results, since patients actively volunteered to participate in this study. This could have resulted in a patient group consisting of patients who live relatively active lives. Fourthly, the technique that was used to define the intensity of psychical activity (LPA, MPA, and VPA) might have been not subtle enough to measure small differences in daily activities. In addition, differences in physical activity (on an individual and group level) could have been so dispersed that generalized parameters did less represent differences in population lifestyles, i.e., DFNA9 patients with less complaints, might compensate with more activity transitions than DFNA9 patients with more complaints.

## 5. Conclusions

This study demonstrated that DFNA9 patients with bilateral vestibulopathy feel significantly more limited during daily activities than age-matched controls, especially regarding social participation. However, this was not reflected in the type of activities, or the amount or type of physical activities performed during daily life. Therefore, self-reported functional limitations and activities in which participants feel limited might be considered as part of candidacy criteria and/or outcome measures for therapeutic interventions. In addition, the amount and classification of physical activities performed during daily life need to be addressed more specifically in future research.

## Figures and Tables

**Table 1:** Characteristics of the DFNA9 patients with BV, including the results of vestibular testing and audiometry.

Patient Number	Gender (M/F)	Age (Years)	vHIT (Gain)		Caloric Test (Sum of Bithermal Max. Peak SPV)		Audiometry (PTA dB HL)	
			Right	Left	Right	Left	Right	Left
1.	M	54	0.56	0.08	-	-	30	40
2.	F	52	0.38	0.38	-	-	70	70
3.	F	50	0.39	0.63	-	-	50	20
4.	M	63	0.03	0.15	-	-	70	60
5.	F	71	0.21	0.47	-	-	-	-
6.	F	61	0.58	0.26	3	3	10	10
7.	F	54	0.28	0.32	3	1	10	10
8.	M	61	-	-	0	0	70	70
9.	F	70	-	-	0	0	-	-
10.	M	75	-	-	0	0	70	70
11.	F	59	0	0	-	-	60	30
12.	F	72	-	-	0	0	-	-
13.	F	66	0.32	0.04	-	-	-	-
14.	F	54	-	-	1	2	30	80
15.	F	64	0.03	0.06	-	-	-	-

M = male; F = female; vHIT = video head impulse test; SPV = slow-phase velocity; PTA = pure tone average (500–4000 Hz); BV= bilateral vestibulopathy; dB HL= decibel (dB) hearing loss (HL).

## Data Availability

The data that support the findings of this study are available from the corresponding author, upon reasonable request.

## Supplementary Materials

The following supporting information can be downloaded at: https://www.mdpi.com/article/10.3390/jcm13041131/s1, S1: DizzyQuest questionnaires.
