# Supplementary material for: The DizzyQuest Combined with Accelerometry: Daily Physical Activities and Limitations among Patients with Bilateral Vestibulopathy Due to DFNA9
Source: J Clin Med. 2024 Feb 17;13(4):1131. doi: 10.3390/jcm13041131 (PMC10889390; doi:10.3390/jcm13041131)
Supplement: Supplementary file 1 [file jcm-13-01131-s001.zip › jcm-1854270-supplementary.pdf]

Supplementary Material S1

DizzyQuest questionnaires

Below the specific set of questionnaires of the within-day and evening questionnaires. For questions about symptoms a seven-point Likert scale is used, ranging from 1 (not at all) to 7 (very).

Within-day questionnaire

Question number	Question	Answer scale	Answer options
1.	I feel relaxed	Likert 7	1 = not at all, 4 = mildly, 7 = very much
2.	I feel cheerful	Likert 7	1 = not at all, 4 = mildly, 7 = very much
3.	I feel irritated	Likert 7	1 = not at all, 4 = mildly, 7 = very much
4.	I feel confident	Likert 7	1 = not at all, 4 = mildly, 7 = very much
5.	I am worrying, about things	Likert 7	1 = not at all, 4 = mildly, 7 = very much
6.	I feel anxious	Likert 7	1 = not at all, 4 = mildly, 7 = very much
7.	I am afraid to fall		
8.	I feel tired	Likert 7	1 = not at all, 4 = mildly, 7 = very much
9.	I generally feel well at the moment	Likert 7	1 = not at all, 4 = mildly, 7 = very much
10.	I am able to concentrate well	Likert 7	1 = not at all, 4 = mildly, 7 = very much
11.	I feel in control	Likert 7	1 = not at all, 4 = mildly, 7 = very much
12.	What am I doing (just before the beep	Multiple choice	Resting, Work/ school, Housekeeping, Hygiene, Eating/ drinking, Active relaxation, Passive relaxation, Something else
13.	I feel I'm being active	Likert 7	1 = not at all, 4 = mildly, 7 = very much
14.	I can do this well	Likert 7	1 = not at all, 4 = mildly, 7 = very much
15.	I'd rather be doing something else	Likert 7	1 = not at all, 4 = mildly, 7 = very much
16.	Where am I?	Multiple choice	At home, at someone else's home, work, school, public place, transport
17.	Who am I with?	Multiple choice (Multiple answers possible)	Partner, family resident, Family non-resident, friends, colleagues, acquaintances, strangers, others, nobody
18.	I like this (social situation)	Likert 7	1 = not at all, 4 = mildly, 7 = very much
19.	I have an attack at this moment	Multiple choice	Yes, No
20.	Since the last BEEP I have had an attack	Multiple choice	Yes, No

21.	Since the last beep I have used	Multiple choice	Caffeine, Nicotine, Alcohol, Medication, Other substance, Tea, Chocolate, Something else, Nothing of these
22.	I now suffer from	Multiple choice	Dizziness, Nausea, Imbalance and/or feeling drunk, Difficulty hearing with 1 or both ears, Ringing in 1 or both ears (tinnitus), Aural fullness, Headache, Light sensitivity, Sound sensitivity, Hypersensitivity for movements and/or hustle and bustle going on around me, Seeing flashes of light and/or zigzag lines, Numbness and/or tingling in arms and/or legs and/or face , Problems with clarity of vision while moving, Difficulty reading, None of the above
23.	This beep bothered me	Likert 7	1 = not at all, 4 = mildly, 7 = very much

<u>Evening- questionnaire</u>

Question number	Question	Answer scale	Answer options
1.	I considered this a normal day	Likert 7	1 = not at all, 4 = mildly, 7 = very much
2.	Overall I felt good today	Likert 7	1 = not at all, 4 = mildly, 7 = very much
3.	Overall I felt tired today	Likert 7	1 = not at all, 4 = mildly, 7 = very much
4.	Overall I felt relaxed today	Likert 7	1 = not at all, 4 = mildly, 7 = very much
5.	Overall I worried a lot today	Likert 7	1 = not at all, 4 = mildly, 7 = very much
6.	Overall I managed to concentrate well today	Likert 7	1 = not at all, 4 = mildly, 7 = very much
7.	Today I experienced dizziness	Likert 7	1 = not at all, 4 = mildly, 7 = very much
8.	Today I experienced nausea	Likert 7	1 = not at all, 4 = mildly, 7 = very much
9.	Today I experience balance problems	Likert 7	1 = not at all, 4 = mildly, 7 = very much
10.	Today I experienced hearing loss on the left hand side	Likert 7	1 = not at all, 4 = mildly, 7 = very much
11.	Today I experienced hearing loss on the right hand side	Likert 7	1 = not at all, 4 = mildly, 7 = very much
12.	Today I experienced tinnitus on the left hand side	Likert 7	1 = not at all, 4 = mildly, 7 = very much
13.	Today I experienced tinnitus on the right hand side	Likert 7	1 = not at all, 4 = mildly, 7 = very much
14.	Today I experienced aural fullness in one or two ears	Likert 7	1 = not at all, 4 = mildly, 7 = very much
15.	Today I experienced headaches	Likert 7	1 = not at all, 4 = mildly, 7 = very much

16.	Today I experienced light hypersensitivity	Likert 7	1 = not at all, 4 = mildly, 7 = very much
17.	Today I experienced sound hypersensitivity	Likert 7	1 = not at all, 4 = mildly, 7 = very much
18.	Today I experienced light flashes, zigzag lines, coloured and/or black dots in my vision	Likert 7	1 = not at all, 4 = mildly, 7 = very much
19.	Today I experienced a strange feeling in my arms, legs and/or face	Likert 7	1 = not at all, 4 = mildly, 7 = very much
20.	Today I experienced problems when I moved	Likert 7	1 = not at all, 4 = mildly, 7 = very much
21.	Today I had difficulty reading	Likert 7	1 = not at all, 4 = mildly, 7 = very much
22.	How many attacks of dizziness, nausea and/or hearing loss did you have today?	Multiple choice	0,1,2,3 or more (if 0, then directly to question 26)
23.	Did the first attack of today already start yesterday?	Multiple choice	Yes/no
24.	The attack(s) was/were triggert by:	Multiple choice (every option possible)	Spontaneous, head- and/or body movement, strong sensations (light, sound, busy environment), stress, something else (open field)
25.	I could not function today due to the attack(s)	Multiple choice	Less than 20 minutes, between 20 minutes and 3 hours, between 3 hours and 12 hours, over 12 hours
26.	How much were you hindered in your daily life	Likert 7	1 = not at all, 4 = mildly, 7 = very much<br>(if 1, then end of questionnaire)
27.	I was mostly hindered in:	Multiple choice (pick one option)	Work/study, household tasks, social engagements, travel, hobbies, sports/movement, sleep
